# DEC1 regulates osteoblast proliferation and differentiation via the RUNX2 signaling pathway: Insights into the molecular mechanism of DEC1-mediated transcriptional regulation in bone metabolism

**DOI:** 10.1515/biol-2025-1217

**Published:** 2026-02-24

**Authors:** Wenjie Qian, Kai Mei, Lei Zhu, Jinpeng Lv, Changjun Yun

**Affiliations:** Department of Orthopedics, Wujin Hospital Affiliated with Jiangsu University, Changzhou, 213002, China; Department of Orthopedics, The Wujin Clinical College of Xuzhou Medical University, Changzhou, 213002, China; Pharmaceutical College, Changzhou University, Changzhou, 213164, China

**Keywords:** DEC1, RUNX2 signaling pathway, osteoblast differentiation, osteoclast activity, bone metabolism

## Abstract

Differentiated embryo chondrocyte 1 (Dec1, protein DEC1) plays a critical role in bone metabolism, but its interaction with the Runt-related transcription factor 2 (RUNX2) signaling pathway remains poorly understood. To investigate how DEC1 regulates osteoblast and osteoclast differentiation through the RUNX2 signaling pathway. To model estrogen deficiency–induced osteoporosis, ovariectomy (OVX) was performed in DEC1 knockout (DEC1^−/−^) and wild-type (DEC1^+/+^) mice, and bone parameters were assessed by micro-computed tomography (micro-CT). Primary bone marrow mesenchymal stem cells (BMSCs) were isolated for osteoblast induction, and RAW 264.7 macrophage-like cells were used for osteoclast differentiation. Flow cytometry was applied to assess lineage markers. RUNX2 expression and transcriptional activity were analyzed by qRT-PCR, Western blotting, GFP-tagging, and chromatin immunoprecipitation sequencing (ChIP-seq). DEC1^−/−^ mice showed significant reductions in bone mineral density (BMD), bone volume fraction (BV/TV), trabecular number (Tb.N), and trabecular thickness (Tb.Th) under OVX. *In vitro*, DEC1 knockdown increased RUNX2 expression but impaired osteoblast differentiation, with reduced ALP, Osterix, and Osteocalcin expression. ChIP-seq confirmed RUNX2 enrichment near transcription start sites (TSS). Osteoclast markers (CD254, TCIRG1, ACP5) were also reduced in DEC1^−/−^ mice. DEC1 regulates bone metabolism by modulating RUNX2 signaling, highlighting its dual role in osteoblast and osteoclast differentiation.

## 1 Introduction

Postmenopausal osteoporosis is a major public health concern, characterized by progressive bone loss and increased fracture risk due to estrogen deficiency [1], [2], [3]. Clinical studies have shown that estrogen plays a critical role in maintaining bone density by balancing bone formation and resorption. Following menopause, the decline in estrogen levels leads to heightened osteoclast activity and reduced osteoblast function, resulting in rapid bone loss, especially in the first few years after menopause [4], 5]. Epidemiological data indicate that nearly one-third of postmenopausal women experience osteoporosis, with fractures of the hip, spine, and wrist being the most common complications [6], 7]. Despite advances in understanding bone biology, the underlying molecular mechanisms of estrogen deficiency-induced bone loss remain incompletely understood, emphasizing the need for further research.

Osteoblasts, the primary bone-forming cells, are critical for bone remodeling and repair. Their proliferation and differentiation are tightly regulated by signaling pathways and transcription factors that ensure proper bone formation [8], 9]. Osteoblast differentiation involves the sequential expression of specific markers, such as alkaline phosphatase (ALP), Osterix, and Osteocalcin [10], [11], [12]. Disruption of osteoblast activity, as observed in estrogen deficiency, exacerbates bone loss and impairs skeletal integrity, highlighting the need to explore regulators of osteoblast function [13], [14], [15], [16].

Differentiated embryo-chondrocyte expressed gene 1 (*Dec1*), a basic helix-loop-helix transcription factor, is involved in various cellular processes, including differentiation, proliferation, and metabolism [17], [18], [19]. Recent studies have shown that DEC1 is expressed in bone-related tissues and may contribute to osteogenic regulation. For instance, reduced DEC1 expression has been linked to diminished osteogenic potential in mesenchymal stem cells, while other work has associated DEC1 with chondrocyte maturation and skeletal development [20]. Despite these observations, the role of DEC1 in the transcriptional control of osteoblast differentiation remains poorly defined. DEC1 is also known to modulate signaling cascades such as hypoxia-inducible factor (HIF) pathways and circadian gene networks, both of which intersect with osteogenic signaling. Moreover, bioinformatic analyses have identified putative DEC1 binding motifs in the promoters of several osteogenic genes, many of which are established targets of RUNX2. Together, these findings provide a strong biological rationale for investigating whether DEC1 regulates bone metabolism through modulation of RUNX2 activity.

RUNX2 (Runt-related transcription factor 2) is a master regulator of osteoblast differentiation and bone formation. It controls the expression of essential osteogenic genes, including ALP, Osterix, and Osteocalcin, which are critical for osteoblast maturation and function [21], [22], [23]. Interestingly, bioinformatic analyses have predicted potential DEC1 binding motifs in the promoters of osteogenic genes that overlap with established RUNX2 targets, suggesting that DEC1 may modulate RUNX2 activity directly or indirectly. This overlap provides a strong biological rationale for investigating DEC1 as an upstream regulator of RUNX2.

This study aims to elucidate the role of DEC1 in regulating osteoblast proliferation and differentiation via the RUNX2 signaling pathway. By employing DEC1 knockout models and cell-based experiments, we explore the molecular mechanisms underlying DEC1-mediated transcriptional regulation, contributing to a deeper understanding of bone metabolism and potential therapeutic targets for osteoporosis.

## 2 Materials and methods

### 2.1 Animal experiments

*Dec1* knockout mice (DEC1^−/−^; RIKEN BioResource Research Center, Tsukuba, Japan, Cat# RBRC05555) were purchased from RIKEN. Wild-type littermates (DEC1^+/+^) were used as controls. In total, 40 female C57BL/6 mice (Charles River Laboratories, Japan, Cat# 000664) were used in this study, with 10 mice allocated to each experimental group (DEC1^−/−^ OVX, DEC1^+/+^ OVX, DEC1^−/−^ sham, and DEC1^+/+^ sham). All mice were housed in a pathogen-free environment under controlled conditions (12-h light/dark cycle, 22 ± 2 °C, and 50–60 % humidity) with free access to food and water. Ovariectomy (OVX) was performed on eight-week-old DEC1^−/−^ and DEC1^+/+^ female mice to induce estrogen-deficient bone loss. Sham surgeries were conducted in control groups for comparison. After 8 weeks, mice were sacrificed, and femurs were harvested for analysis.

**Ethical approval:** The research related to animal use has been complied with all the relevant national regulations and institutional policies for the care and use of animals, and has been approved by the Ethics Committee of Wujin Clinical College of Xuzhou Medical University.

### 2.2 Micro-CT analysis

Bone microarchitecture was assessed using high-resolution micro-computed tomography (micro-CT; SkyScan 1176, Bruker, Kontich, Belgium). Femurs were fixed in 4 % paraformaldehyde for 24 h and stored in 70 % ethanol until scanning. Scans were performed at 9 μm voxel resolution, 50 kV tube voltage, 500 μA current, and with a 0.5 mm aluminum filter. Each femur was scanned along the longitudinal axis with a 0.7° rotation step over 180°. Three-dimensional reconstruction and quantitative analysis were carried out using NRecon and CTAn software (Bruker). A standardized volume of interest (VOI) was defined in the distal femoral metaphysis, beginning 0.5 mm proximal to the growth plate and extending proximally for 1.0 mm. Trabecular bone parameters were calculated, including bone mineral density (BMD), bone volume fraction (BV/TV), trabecular number (Tb.N), and trabecular thickness (Tb.Th).

### 2.3 Cell culture and differentiation

RAW 264.7 cells (murine macrophage-like cell line, ATCC^®^ TIB-71™, American Type Culture Collection, Manassas, VA, USA) were used in this study. This cell line, derived from an Abelson murine leukemia virus–induced tumor in BALB/c mice, is widely employed as a model for osteoclast differentiation due to its ability to form multinucleated osteoclast-like cells upon receptor activator of nuclear factor κB ligand (RANKL) stimulation. RAW 264.7 cells were cultured in α-MEM (Gibco, Thermo Fisher Scientific, Cat# 12561056) supplemented with 10 % fetal bovine serum (FBS; Gibco, Cat# 10099141C) and 1 % penicillin-streptomycin (Gibco, Cat# 15140122), and maintained at 37 °C in a humidified atmosphere with 5 % CO_2_.

DEC1 knockout in RAW 264.7 cells was achieved using a CRISPR/Cas9-based gene editing approach. Specifically, a plasmid containing DEC1-targeting single guide RNA (sgRNA) sequences and Cas9 nuclease (PX459 vector, Addgene, Cat# 62988) was constructed. Cells were transfected with the CRISPR/Cas9 plasmid using Lipofectamine 3000 (Thermo Fisher Scientific, Cat# L3000008) following the manufacturer’s instructions. Successfully transfected clones were selected using puromycin (2 μg/mL; Sigma-Aldrich, Cat# P8833), and knockout efficiency was validated by Western blotting and qPCR. For osteoclast differentiation, RAW 264.7 cells were plated at a density of 5 × 10^4^ cells per well in 6-well plates and treated with recombinant mouse RANKL (PeproTech, Cat# 315-11) at 50 ng/mL for 5 days. The culture medium was replaced every 48 h.

### 2.4 Isolation and culture of bone marrow mesenchymal stem cells (BMSCs)

Bone marrow mesenchymal stem cells (BMSCs) were isolated from the femurs and tibias of DEC1^−/−^ and DEC1^+/+^ mice (8 weeks old). Briefly, mice were euthanized, and femurs and tibias were aseptically dissected. The bone marrow cavity was flushed with complete α-MEM (Minimum Essential Medium, α-MEM; Gibco, Cat# 12571-063) supplemented with 10 % fetal bovine serum (FBS; Gibco, Cat# 10099141), and 1 % penicillin–streptomycin (Thermo Fisher Scientific, Cat# 15140122). The cell suspension was filtered through a 70 μm cell strainer (Corning, Cat# 352350) to remove debris and centrifuged at 300 × *g* for 5 min. The pellet was resuspended and plated in T-75 flasks. After 24 h, non-adherent hematopoietic cells were removed by medium replacement. The adherent fibroblast-like cells were expanded and cultured until passage 2 for subsequent experiments.

Osteogenic differentiation was induced using osteogenic induction medium containing 50 μg/mL ascorbic acid (Sigma-Aldrich, Cat# A4403), 10 mM β-glycerophosphate (Sigma-Aldrich, Cat# G9422), and 10 nM dexamethasone (Sigma-Aldrich, Cat# D4902). After 14 days, differentiation efficiency was assessed by analyzing the expression of osteogenic markers, including alkaline phosphatase (ALP), Osterix, and Osteocalcin, as well as mesenchymal stem cell markers CD90 and CD105.

### 2.5 qRT-PCR

Total RNA was extracted from cultured cells or mouse bone tissues using TRIzol Reagent (Invitrogen, Thermo Fisher Scientific, Cat# 15596026) following the manufacturer’s protocol. RNA concentration and purity were determined using a NanoDrop 2000 spectrophotometer (Thermo Fisher Scientific). Reverse transcription was performed using the PrimeScript RT Reagent Kit (Takara Bio, Cat# RR037A). Quantitative real-time PCR was conducted using SYBR Green PCR Master Mix (Applied Biosystems, Thermo Fisher Scientific, Cat# 4309155) on a StepOnePlus Real-Time PCR System (Applied Biosystems, Thermo Fisher Scientific).

Primer sequences were designed to specifically amplify genes of interest, including cytokines (TNF-α, IFN-γ, IL-1β, IL-6), osteoblast markers (ALP, Osterix, Osteocalcin), and RUNX2. GAPDH was used as the internal control. Relative expression levels were calculated using the 2^ˆ−ΔΔCt^ method, and all reactions were performed in triplicate.

### 2.6 ChIP-seq

Chromatin immunoprecipitation (ChIP) was performed to identify RUNX2 binding sites. Cells were crosslinked with 1 % formaldehyde (Sigma-Aldrich, Cat# F8775) for 10 min at room temperature and quenched with 0.125 M glycine (Sigma-Aldrich, Cat# G7126). Chromatin was fragmented to 200–500 bp by sonication using a Bioruptor Pico sonicator (Diagenode). Immunoprecipitation was carried out using anti-RUNX2 antibody (Cell Signaling Technology, Cat# 12556) and protein A/G magnetic beads (Thermo Fisher Scientific, Cat# 88803).

Immunoprecipitated DNA was purified using a MinElute PCR Purification Kit (Qiagen, Cat# 28006) and used to construct sequencing libraries with the NEBNext Ultra II DNA Library Prep Kit (New England Biolabs, Cat# E7645). Sequencing was performed on an Illumina NovaSeq 6000 platform (Illumina, San Diego, CA, USA). Raw reads were aligned to the mouse reference genome (mm10) using Bowtie2, and peak enrichment near transcription start sites (TSS) was analyzed using HOMER software (version 4.11) to determine RUNX2 binding patterns.

### 2.7 GFP-tagged RUNX2

GFP-tagged RUNX2 plasmids were transfected into BMSCs using Lipofectamine 3000 according to the manufacturer’s instructions. After 48 h, GFP fluorescence was observed under a confocal laser scanning microscope to evaluate RUNX2 localization and binding at transcription start sites (TSS). Quantification of GFP signals was performed using ImageJ software to assess binding intensity.

### 2.8 Flow cytometry

RAW 264.7 cells and bone marrow mesenchymal stem cells (BMSCs) were harvested and stained with specific fluorescently labeled antibodies targeting differentiation markers. Osteoclast markers included CD254 (RANKL, Abcam, Cat# ab45039), CD265 (RANK, BioLegend, Cat# 113803), TCIRG1 (Santa Cruz Biotechnology, Cat# sc-271943), ACP5 (TRAP, Novus Biologicals, Cat# NBP1-69870), MMP9 (Abcam, Cat# ab38898), and ITGB3 (Integrin β3, BD Biosciences, Cat# 553347). Osteoblast markers included CD90 (BioLegend, Cat# 140302) and CD105 (eBioscience/Thermo Fisher Scientific, Cat# 12-1057-42).

Stained cells were analyzed using a flow cytometer (BD LSRFortessa™, BD Biosciences, San Jose, CA, USA), and data were processed using FlowJo software (Version 10.8.1, BD Biosciences) to compare marker expression between DEC1^−/−^ and DEC1^+/+^ groups.

### 2.9 Western blotting

Protein expression levels were analyzed by Western blotting. Cells or bone tissue samples were lysed in RIPA buffer (Beyotime, Cat# P0013B) supplemented with protease and phosphatase inhibitors (Roche, Cat# 11873580001). Protein concentrations were determined using the BCA Protein Assay Kit (Thermo Fisher Scientific, Cat# 23227). Equal amounts of protein (20–30 μg) were separated by 10 % SDS–PAGE and transferred onto PVDF membranes (Millipore, Cat# IPVH00010). Membranes were blocked with 5 % non-fat milk in TBST buffer for 1 h at room temperature and incubated overnight at 4 °C with primary antibodies against RUNX2 (Cell Signaling Technology, Cat# 12556, 1:1,000 dilution) and GAPDH (Cell Signaling Technology, Cat# 5174, 1:2,000 dilution). After washing, membranes were incubated with HRP-conjugated secondary antibodies (Cell Signaling Technology, Cat# 7074, 1:5,000 dilution) for 1 h at room temperature. Protein bands were visualized using an enhanced chemiluminescence (ECL) detection kit (Thermo Fisher Scientific, Cat# 32106) and imaged with a ChemiDoc imaging system (Bio-Rad). Densitometric analysis was performed using ImageJ software, with GAPDH serving as the internal loading control.

### 2.10 Alizarin Red S staining

Bone marrow mesenchymal stem cells (BMSCs) isolated from DEC1^+/+^ and DEC1^−/−^ mice were seeded into 6-well plates at a density of 5 × 10^4^ cells/well and cultured in osteogenic induction medium containing 50 μg/mL ascorbic acid (Sigma-Aldrich, Cat# A4403), 10 mM β-glycerophosphate (Sigma-Aldrich, Cat# G9422), and 10 nM dexamethasone (Sigma-Aldrich, Cat# D4902) for 14 days, with the medium refreshed every 2–3 days. At the end of induction, cells were washed twice with PBS and fixed with 4 % paraformaldehyde (Sigma-Aldrich, Cat# 158127) for 30 min at room temperature. Following fixation, cells were rinsed with distilled water and stained with 2 % Alizarin Red S solution (pH 4.2; Sigma-Aldrich, Cat# A5533) for 20 min at room temperature with gentle shaking. Excess dye was removed by thorough washing with distilled water until the background was clear. Mineralized calcium nodules were visualized under a light microscope (Olympus, Japan), and representative images were captured. For quantitative analysis, bound dye was solubilized using 10 % (w/v) cetylpyridinium chloride (Sigma-Aldrich, Cat# C0732), and absorbance was measured at 562 nm using a microplate reader (BioTek, USA).

### 2.11 Statistical analysis

All experiments were performed at least three times independently. Data were presented as mean ± standard deviation (SD). Statistical analysis was conducted using GraphPad Prism software (Version 9.5.1, GraphPad Software, San Diego, CA, USA). Differences between groups were analyzed by one-way ANOVA followed by Tukey’s post hoc test for multiple comparisons. Statistical significance was defined as *p* < 0.05, with significance levels indicated as ns (not significant), *p < 0.05, **p < 0.01, and *p < 0.001.

## 3 Results

### 3.1 Establishment of the DEC1 knockout mouse model and OVX-induced bone loss

**Figure 1:**
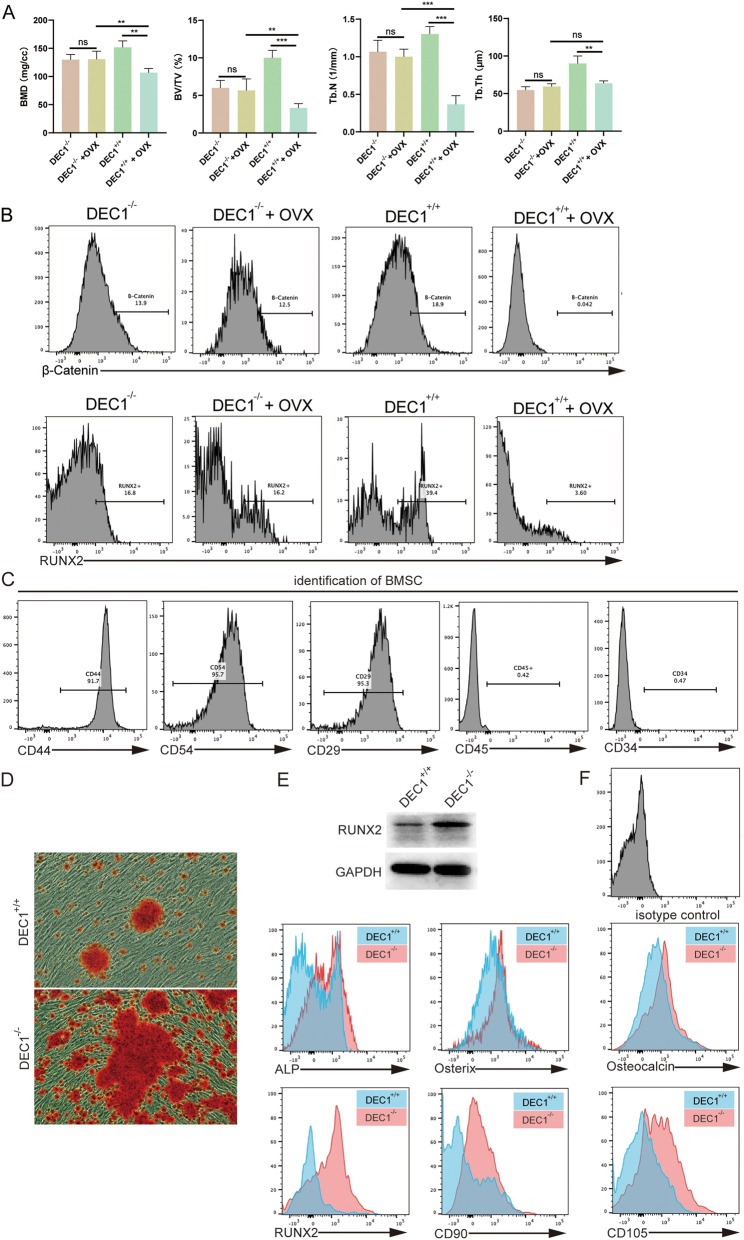
DEC1 regulates osteogenesis and protects against OVX-induced bone loss. (A) Micro-CT analysis of bone parameters: bone mineral density (BMD), bone volume fraction (BV/TV), trabecular number (Tb.N), and trabecular thickness (Tb.Th). Data are presented as mean ± SD, with *n* = 10 mice per group. Statistical significance is denoted as ns (not significant), *p < 0.05, **p < 0.01, and ***p < 0.001. OVX significantly exacerbated bone loss in DEC1^−/−^ mice compared to DEC1^+/+^ controls. (B) Representative flow cytometry histograms showing β-catenin (top row) and RUNX2 (bottom row) expression in bone marrow-derived cells from DEC1^−/−^ and DEC1^+/+^ mice with or without ovariectomy (OVX). In wild-type mice, OVX markedly decreased expression of both markers. In contrast, DEC1-deficient mice showed blunted responses to OVX, with relatively preserved RUNX2 levels and attenuated suppression of β-catenin. Data represent mean ± SD from three independent experiments. (C) Identification of BMSC surface markers by flow cytometry. BMSCs exhibited positive expression of CD29, CD44, and CD54, and negative expression of CD45 and CD34. Data are representative of three independent experiments. (D) Alizarin Red S staining of BMSCs after osteogenic induction for 14 days. DEC1^−/−^ BMSCs exhibited significantly more extensive calcium nodule deposition compared to DEC1^+/+^ controls. (E) Western blot validation of RUNX2 expression in bone marrow–derived cells from DEC1^+/+^ and DEC1^−/−^ mice. GAPDH was used as the loading control. (F) Expression of osteogenic markers (ALP, Osterix, Osteocalcin, RUNX2, CD90, CD105) after osteogenic induction in DEC1^+/+^ and DEC1^−/−^ mice. DEC1^−/−^ mice exhibited higher expression levels of these markers, indicating enhanced osteogenic induction potential. Data are presented as mean ± SD from three independent experiments. An isotype control was included to exclude autofluorescence and nonspecific antibody binding, and marker positivity was determined relative to this control.

To investigate the role of DEC1 in bone loss, DEC1^−/−^ mice were used to model gene knockout effects, and ovariectomy (OVX) was performed to mimic estrogen deficiency-induced bone loss. Micro-CT analysis was conducted to evaluate bone parameters, including bone mineral density (BMD), bone volume fraction (BV/TV), trabecular number (Tb.N), and trabecular thickness (Tb.Th) (Figure 1A). Under sham conditions, no significant differences in BMD, BV/TV, Tb.N, or Tb.Th were observed between DEC1^−/−^ and DEC1^+/+^ mice. However, following OVX, DEC1^−/−^ mice exhibited significantly reduced BMD, BV/TV, Tb.N, and Tb.Th compared to DEC1^+/+^ mice. OVX treatment further exacerbated bone loss in DEC1^−/−^ mice, as shown by the statistical comparisons (ns, **p < 0.01, ***p < 0.001).

Flow cytometric analysis revealed distinct regulatory patterns of β-catenin and RUNX2 expression across *Dec1* genotypes and hormonal states. β-catenin, a key mediator of the canonical Wnt signaling pathway that promotes osteoblast differentiation and bone formation, was also evaluated in bone marrow–derived cells. In wild-type (DEC1^+/+^) mice, ovariectomy (OVX) markedly suppressed both β-catenin and RUNX2 expression, indicating a strong dependence of these osteogenic factors on estrogen signaling. In contrast, DEC1-deficient (DEC1^−/−^) mice exhibited reduced baseline expression of β-catenin but maintained relatively stable RUNX2 expression regardless of OVX treatment (Figure 1B). Thus, DEC1^+/+^ mice displayed higher baseline RUNX2 expression in bone marrow-derived cells compared to DEC1^−/−^ mice.

To further confirm the role of DEC1 in osteogenic differentiation, flow cytometry was used to identify bone marrow mesenchymal stem cell (BMSC) surface markers (Figure 1C). DEC1^+/+^ BMSCs exhibited a typical phenotype characterized by positive expression of CD29, CD44, and CD54, and negative expression of CD45 and CD34.

Functional validation with Alizarin Red S staining demonstrated markedly increased mineralized nodule formation in DEC1^−/−^ BMSCs compared to DEC1^+/+^ controls, indicating enhanced osteogenic differentiation capacity in the absence of DEC1 (Figure 1D).

Furthermore, osteogenic differentiation assays revealed that DEC1^−/−^ mice exhibited significantly elevated expression levels of osteogenic markers, including ALP, Osterix, Osteocalcin, and RUNX2, as well as mesenchymal stem cell markers CD90 and CD105, compared to DEC1^+/+^ mice. These results were confirmed by both flow cytometry and Western blot analysis, indicating that DEC1 knockout enhances osteogenic induction potential (Figure 1E and F).

### 3.2 DEC1 knockdown promotes RUNX2 expression, which subsequently inhibits osteoblast differentiation

**Figure 2:**
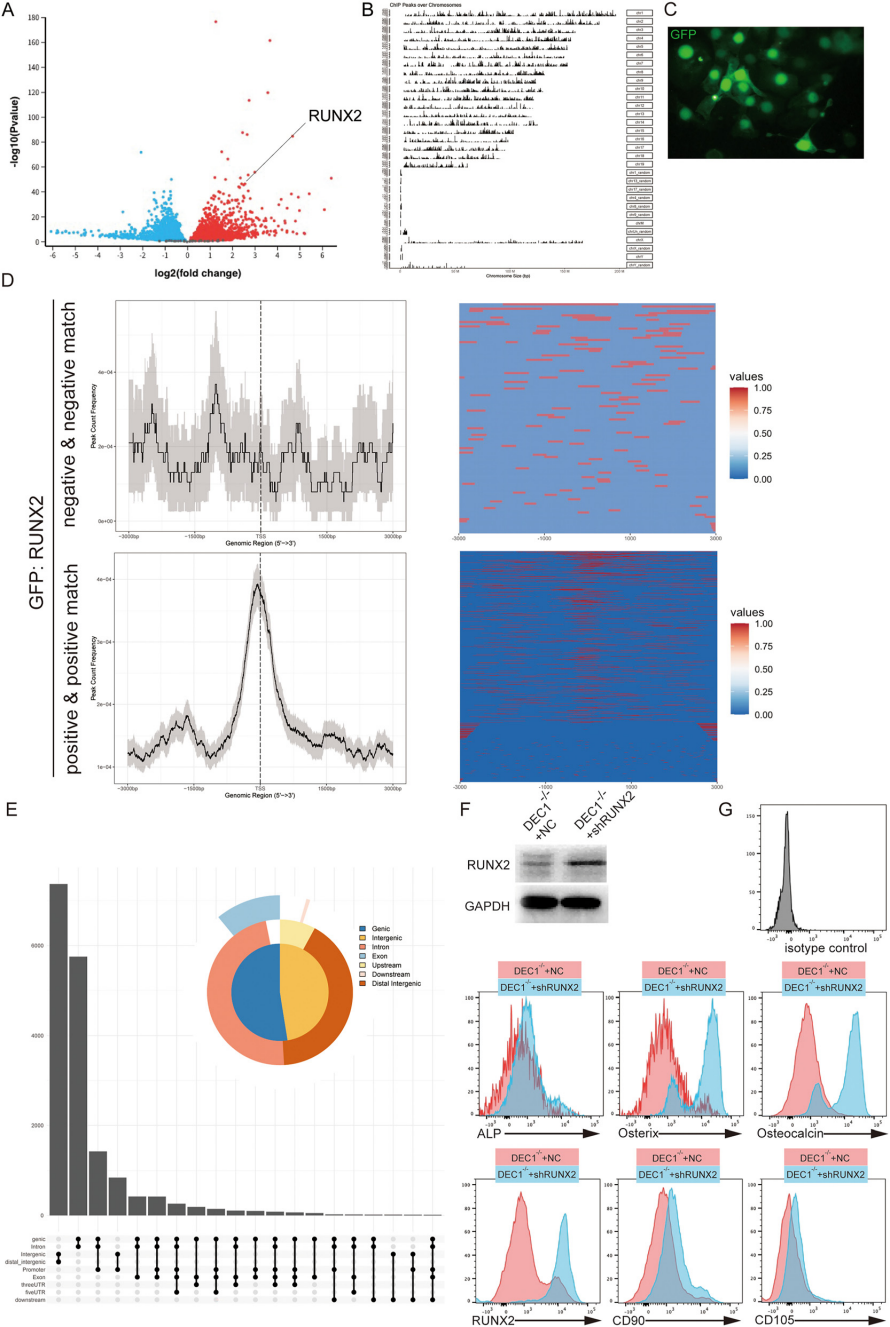
DEC1 suppresses RUNX2 and inhibits osteoblast differentiation. (A) Volcano plot showing significant upregulation of RUNX2 expression following DEC1 knockdown. The highlighted point indicates RUNX2. (B) ChIP-seq analysis of RUNX2 binding sites, with peaks enriched near transcription start sites (TSS). (C and D) Analysis of RUNX2 binding patterns using GFP-tagged RUNX2. After 48 h of transfection, GFP fluorescence was observed under a confocal laser scanning microscope, showing nuclear localization of RUNX2-GFP. Binding signals were subsequently evaluated in “Negative & Negative Match” and “Positive & Positive Match” groups, and heatmaps displayed the intensity of RUNX2 binding near transcription start sites (TSS) regions. (E) Distribution of RUNX2 binding site types, including promoter and intronic regions. (F) Western blot analysis of RUNX2 protein levels in DEC1^−/−^ + NC and DEC1^−/−^ + shRUNX2 groups, with GAPDH as the loading control. RUNX2 levels were markedly reduced in the shRUNX2 group. (G) Expression levels of osteogenic markers (ALP, Osterix, Osteocalcin, RUNX2, CD90, CD105) in DEC1^−/−^ + NC and DEC1^−/−^ + shRUNX2 groups during osteoblast induction. Data are presented as mean ± SD from three independent experiments. Histograms show that the DEC1^−/−^ + shRUNX2 group exhibited reduced expression levels of these markers compared to the DEC1^−/−^ + NC group. An isotype control was included to exclude autofluorescence and nonspecific antibody binding, and positivity was determined relative to this control.

A volcano plot revealed significant upregulation of RUNX2 expression following DEC1 knockdown (Figure 2A), with RUNX2 showing a log2 (fold change) of 3.5. These findings suggest that RUNX2 plays a pivotal role in DEC1-mediated regulation. ChIP-seq analysis was performed to investigate RUNX2 binding patterns, revealing that binding peaks were predominantly enriched around transcription start sites (TSS) (Figure 2B). GFP-tagged RUNX2 was utilized to visualize binding specificity, and heatmaps illustrated RUNX2 binding intensity at TSS regions in both “Negative & Negative Match” and “Positive & Positive Match” groups (Figure 2C and D). Analysis of RUNX2 binding site types showed diverse binding across promoter, intronic, and other genomic regions (Figure 2E).

Osteoblast induction experiments demonstrated that compared to the DEC1^−/−^ + NC group, the DEC1^−/−^ + shRUNX2 group exhibited significantly reduced expression levels of osteogenic markers, including ALP, Osterix, Osteocalcin, RUNX2, CD90, and CD105. Western blot analysis confirmed that RUNX2 expression was markedly decreased in the DEC1^−/−^ + shRUNX2 group. Flow cytometry further supported these findings, showing consistent reductions across osteogenic markers. Together, these results support RUNX2 as a positive regulator of osteoblast differentiation and indicate that DEC1 may influence osteogenesis indirectly through modulation of RUNX2 activity (Figure 2F and G).

### 3.3 DEC1 knockout impairs osteoclast differentiation and cytokine production

**Figure 3:**
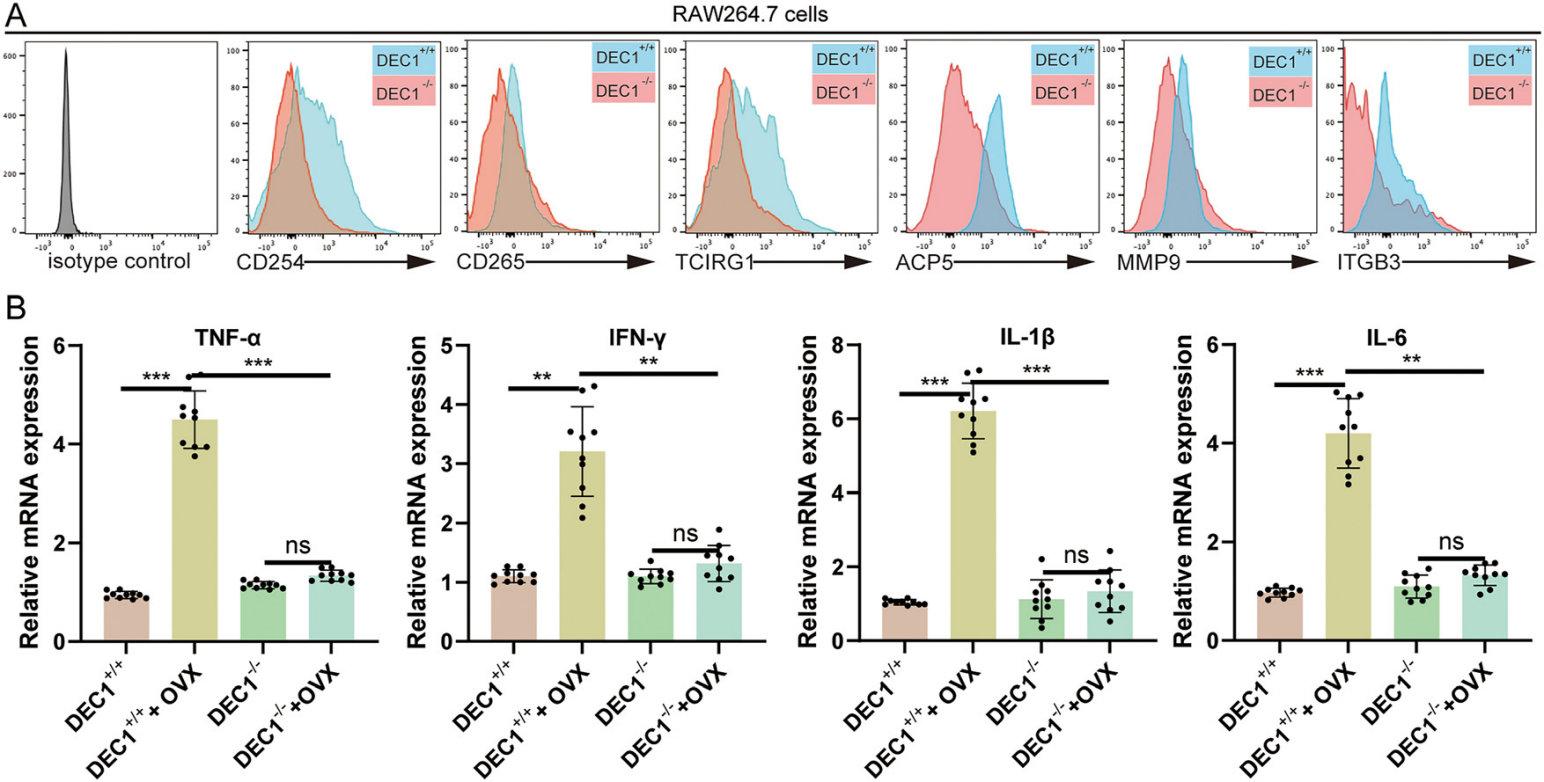
DEC1 knockout reduces osteoclast differentiation in RAW 264.7 cells. (A) Flow cytometry analysis of osteoclast differentiation markers, including CD254, CD265, TCIRG1, ACP5, MMP9, and ITGB3, in RAW 264.7 cells after RANKL stimulation. Data are presented as mean ± SD from three independent experiments. DEC1^−/−^ cells exhibited significantly lower marker expression compared to DEC1^+/+^ cells, indicating impaired osteoclast differentiation. An isotype control was included to exclude autofluorescence and nonspecific antibody binding, and marker positivity was determined relative to this control. (B) Bar graphs showing the relative mRNA expression of cytokines (TNF-α, IFN-γ, IL-1β, IL-6) in bone tissues from DEC1^+/+^ and DEC1^−/−^ mice under normal and OVX conditions. Each group consisted of *n* = 10 mice, and data are presented as mean ± SD. Individual data points from each animal are shown together with error bars. Cytokine expression was significantly reduced in DEC1^−/−^ mice compared to DEC1^+/+^ controls, whereas OVX treatment in DEC1^+/+^ mice further elevated cytokine levels. Statistical significance is indicated as ns (not significant), *p < 0.05, **p < 0.01, and ***p < 0.001.

To examine the role of DEC1 in osteoclast differentiation, RAW 264.7 cells were stimulated with RANKL and analyzed by flow cytometry. DEC1^−/−^ cells exhibited significantly reduced expression of key osteoclast markers, including CD254, CD265, TCIRG1, ACP5, MMP9, and ITGB3, compared to DEC1^+/+^ cells (Figure 3A). These results indicate that DEC1 is essential for proper osteoclast differentiation. Furthermore, cytokine mRNA levels were quantified, showing significantly lower expression of TNF-α, IFN-γ, IL-1β, and IL-6 in DEC1^−/−^ cells compared to DEC1^+/+^ cells under the same conditions (Figure 3B). These findings suggest that DEC1 regulates cytokine production, which is critical for osteoclastogenesis.

## 4 Discussion

This study investigates the role of DEC1 in regulating osteoblast and osteoclast differentiation through the RUNX2 signaling pathway, uncovering its impact on bone metabolism in the context of estrogen deficiency. The findings reveal that DEC1 plays a dual role in bone homeostasis by modulating osteogenic and osteoclastic processes, offering new insights into the molecular mechanisms underlying bone remodeling.

Ovariectomy (OVX) is a well-established model to simulate postmenopausal bone loss caused by estrogen deficiency. DEC1^−/−^ mice exhibited significantly lower bone mineral density (BMD) and compromised trabecular structure compared to DEC1^+/+^ controls. However, following OVX, the reduction in BMD was more pronounced in DEC1^+/+^ mice than in DEC1^−/−^ mice, indicating that DEC1 deficiency confers a relative protective effect against estrogen deficiency–induced bone degeneration. These results align with previous studies demonstrating that estrogen deficiency accelerates bone resorption and impairs bone formation. Our findings uniquely highlight the role of DEC1 in this process, suggesting that while DEC1 contributes to baseline bone homeostasis, its absence may mitigate the skeletal deterioration associated with hormonal imbalance.

Osteoblast differentiation is orchestrated by transcription factors such as RUNX2, which governs the expression of key osteogenic markers, including ALP, Osterix, and Osteocalcin. Previous studies have identified RUNX2 as a master regulator of osteogenesis, but its upstream regulation remains incompletely understood. Our results demonstrate that DEC1 knockdown significantly upregulates RUNX2 expression and enhances its binding activity near transcription start sites (TSS). However, paradoxically, DEC1 knockout mice exhibited increased osteogenic marker expression, indicating enhanced osteoblast differentiation potential in the absence of DEC1. This apparent contradiction may reflect a regulatory feedback mechanism, where excessive or dysregulated RUNX2 activity resulting from DEC1 loss disrupts the fine-tuned balance required for osteoblast function. This is supported by our data showing that suppressing RUNX2 in DEC1^−/−^ cells (DEC1^−/−^ + shRUNX2) reduces osteogenic marker expression. These findings suggest that DEC1 modulates RUNX2 activity to ensure optimal osteoblast differentiation, a mechanism that aligns with reports of transcriptional repressors maintaining balance in RUNX2-mediated osteogenesis.

Osteoclast differentiation, driven by RANKL signaling, is a critical component of bone resorption. DEC1^−/−^ RAW 264.7 cells exhibited significantly reduced expression of osteoclast markers, including CD254, TCIRG1, and MMP9, compared to DEC1^+/+^ cells, suggesting impaired osteoclastogenesis. Additionally, cytokine production, which plays a pivotal role in osteoclast activity, was markedly reduced in DEC1^−/−^ cells, as evidenced by lower levels of TNF-α, IFN-γ, IL-1β, and IL-6. These findings contrast with previous studies emphasizing the pro-inflammatory role of cytokines in promoting osteoclast differentiation, highlighting DEC1 as a potential regulator of inflammatory signaling in bone metabolism. While DEC1’s involvement in cytokine regulation has been studied in other contexts, such as cancer and immune response, its role in osteoclastogenesis is less well understood. Our findings suggest that DEC1 may act upstream of cytokine-mediated osteoclast activity, offering a new perspective on how transcription factors influence the coupling of bone resorption and formation.

Previous studies have largely focused on RUNX2 as the central transcription factor in osteoblast differentiation, with limited exploration of how upstream regulators like DEC1 influence its activity. This study bridges that gap by demonstrating DEC1’s dual role in osteoblast and osteoclast regulation. Unlike prior research that has primarily investigated DEC1 in cancer or circadian biology, our findings establish its importance in bone metabolism. Additionally, while most studies emphasize the anabolic role of RUNX2, our results reveal the complexity of its regulation by DEC1, highlighting a potential inhibitory mechanism required for balanced osteogenesis. In contrast to studies showing DEC1’s role as a general promoter of cellular differentiation, our results reveal a context-dependent regulatory function in bone cells. This divergence underscores the need to further explore how DEC1 interacts with other signaling pathways, such as Wnt/β-catenin, which may synergize with RUNX2 to mediate osteoblast and osteoclast dynamics, and whether DEC1 loss triggers compensatory mechanisms that blunt OVX-induced bone loss.

This study highlights DEC1 as a critical regulator of bone metabolism, with distinct roles in osteoblast and osteoclast differentiation mediated through the RUNX2 signaling pathway. These findings provide a foundation for future research exploring DEC1 as a therapeutic target for osteoporosis and other bone-related disorders. Investigating the crosstalk between DEC1 and other pathways, such as Wnt/β-Catenin or NF-κB, could further elucidate the complex regulatory networks underlying bone homeostasis. Additionally, *in vivo* studies focusing on the temporal and spatial expression of DEC1 during bone remodeling would provide deeper insights into its functional dynamics.
